# Global time-size distribution of volcanic eruptions on Earth

**DOI:** 10.1038/s41598-018-25286-y

**Published:** 2018-05-01

**Authors:** Paolo Papale

**Affiliations:** Istituto Nazionale di Geofisica e Vulcanologia, Sezione di Pisa, via Della Faggiola 32, 56126 Pisa, Italy

## Abstract

Volcanic eruptions differ enormously in their size and impacts, ranging from quiet lava flow effusions along the volcano flanks to colossal events with the potential to affect our entire civilization. Knowledge of the time and size distribution of volcanic eruptions is of obvious relevance for understanding the dynamics and behavior of the Earth system, as well as for defining global volcanic risk. From the analysis of recent global databases of volcanic eruptions extending back to more than 2 million years, I show here that the return times of eruptions with similar magnitude follow an exponential distribution. The associated relative frequency of eruptions with different magnitude displays a power law, scale-invariant distribution over at least six orders of magnitude. These results suggest that similar mechanisms subtend to explosive eruptions from small to colossal, raising concerns on the theoretical possibility to predict the magnitude and impact of impending volcanic eruptions.

## Introduction

Cataclysmic eruptions are, fortunately, rare events, although less rare than the impact from a large celestial body, the latter representing the only other natural event thought to be capable of comparable destruction^1,2^. Less extreme eruptions are by far more frequent, and have repeatedly affected humans during our short history. Large eruptions have been devastating, triggering years-long variations of global climate, and distressing regions as large as Europe causing severe famine, uncounted deaths, and massive migration flows^3–6^.

The difficulty to obtain a quantitative description of the time-size distribution of volcanic eruptions stems from a number of factors: i) the attempts to construct global volcanic databases are only recent^7,8^; ii) such databases reveal a striking deterioration of information with age^9–12^; and iii) with increasing size and rareness of eruptions, the required catalogue completeness extends back to millions of years^8–11^.

Here I reconstruct the global time-size distribution of volcanic eruptions on Earth, starting from the two existing large databases constituted by the Global Volcanism Project (GVP) Holocene eruptions database of the Smithsonian Institution (http://volcano.si.edu), and the Large Magnitude Explosive Volcanic Eruptions (LaMEVE) database realized in the frame of the VOGRIPA project (http://www.bgs.ac.uk/vogripa). These databases, extensively analyzed elsewhere^8–13^, do not include ocean floor-forming eruptions and flood basalt eruptions, that are therefore excluded from the present analysis. In order to classify volcanic eruptions into different size I refer to the VEI scale^14^, reported in both GVP and LaMEVE databases, and of widespread use among volcanologists. The VEI scale combines a series of parameters to define an eruption magnitude, and provides a direct relationship with the volume of erupted magma, which varies with VEI according to a logarithmic scale to account for the many orders of magnitude variations from small up to colossal eruptions (Table 1). The Methods include a discussion on the use of the VEI scale to characterize eruption size, compared to the use of the Magnitude scale^15^ adopted by other authors^11,15–17^. There are 9517 individual eruptions with reported VEI in the joined GVP+LaMEVE database (from here on, simply referred to as the database, described in the Methods), spanning the entire VEI scale over a time extending back to more than 2 Ma BP (millions of years Before Present, conventionally identified with 1950 AD). According to the global objectives of the two databases, individual eruptions often represent an eruptive period lasting up to years, characterized in the database by its most energetic phase (or its highest VEI). To make a comparison with earthquakes, the database would refer to just main shocks, neglecting the detailed description of pre- and post-shock sequences. The database, and consequently the present analysis, is therefore not suited for inspecting behaviors at individual volcano scale, as it refers instead to the global Earth scale.

**Table 1:** . The VEI scale^14^, adapted.

VEI	Volume of erupted magma (m^3^)	Description
0	<10^4^	Gentle, effusive
1	>10^4^	
2	>10^6^	Explosive
3	>10^7^	
4	>10^8^	Explosive: Cataclysmic, Paroxysmal, Colossal
5	>10^9^	
6	>10^10^	
7	>10^11^	
8	>10^12^	

Previous analyses^18–22^ suggest that regional or global eruption rates may have varied in the past tens or hundreds of thousands years, in connection with large-scale climate changes and associated Earth crust loading/unloading by ice or water covers. However, the analysis of the LaMEVE database suggests that apparent eruption rate changes mostly reflect deterioration of information with age, rather than real variations^9^. Accordingly, stationary behavior of Earth at the global scale in relation to volcanic eruption occurrence during last few million years is usually assumed^11,17^.

Previous authors^9–12,16,17^ have outlined possible systematic errors in Magnitude and VEI assignments, e.g., selective VEI, M underestimates due to erosion of volcanic deposits or to difficult estimates of the proportion of erupted material captured by atmospheric circulation and lately dispersed over exceedingly large areas; and have stressed the relevance of possible eruption under-recording. Here I refer to the existing database described above, that is taken to represent the international standard. The present investigation intentionally does not contemplate acting on the database to modify it based on assumed or inferred wrong VEI assignments. Accordingly, the results describe the present level of knowledge as it is embedded in current databases forming state-of-the-art international references. Future database improvements are expected to reflect progress in quantifying past volcanic eruptions and their deposits, likely leading to more robust database and next analyses. However, sensitivity analyses, described in the Methods section, are conducted in order to evaluate the robustness of major conclusions to possible systematic biases in the database.

## Results

**Figure 1:**
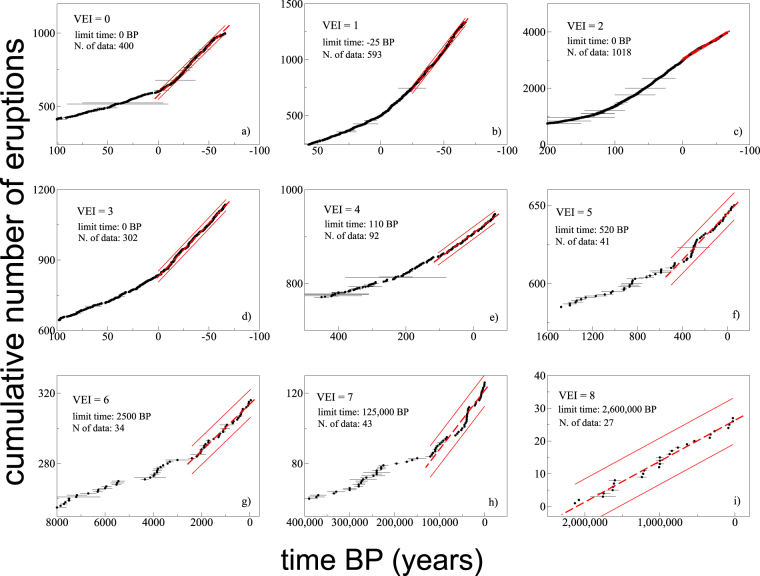
Cumulative plots of the number of eruptions with time for each individual VEI class. The plots represent a zoom in a (relatively) recent time region where approximately linear trends emerge. The horizontal axis refers to years Before Present (BP), conventionally set to zero at year AD 1950. Best-fit linear trends are represented by the thick red dashed lines, while the thin solid red lines bound the 95% confidence band for expected statistical variability of data^23^. Wider confidence limits reflect lower number of data. Each panel reports the time limit of the linear region, which generally increases with increasing VEI, and the number of eruptions in such a region, which generally decreases with increasing VEI.

Although the information in the database significantly deteriorates with age, volcanic eruptions offer the convenient condition that small eruptions, poorly or not preserved in the geological record, are highly frequent and therefore highly represented in the most recent, more complete portion of the database; whereas large eruptions, although rare, are well preserved in the geological record and can be identified back in time and reconstructed with the instruments of geological analysis. That situation is displayed in Fig. 1, where the cumulative distributions of different VEI eruptions show approximately linear trends with time within a relatively recent time window. Similar approximately linear regions for different Magnitude classes of eruptions have been employed^17^ to determine the time periods back in the past over which the assumption of complete eruption recording may be justified. The length of such approximately linear regions, represented in Fig. 1 by red dashed best fit lines, increases with increasing VEI class, as a consequence of both lower frequency and increased preservation of the information with increasing eruption size^11,17^. The number of events recorded in such approximately linear regions depends on the average return time of different VEI eruptions, ranging from several hundreds for small VEI eruptions back to only AD 1950, to less than 30 in slightly more than 2 Ma for the colossal VEI 8 eruptions. The total number of eruptions with assigned VEI that are distributed around the linear trends in Fig. 1 is 2550.

**Figure 2:**
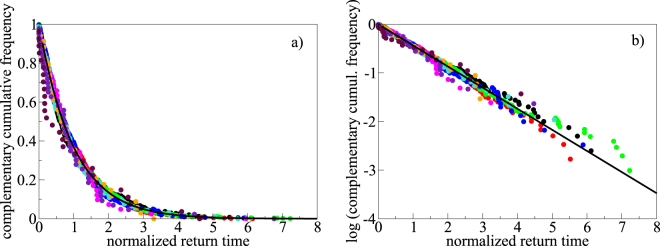
Exponential distribution of return times. (**a**) Complementary cumulative frequency distribution of normalized return times, for eruptions of any VEI from present time (mid-September 2017) back to the limit time reported, for each VEI class, in the panels of Fig. 1. (**b**) Same plot as for panel a, on a logarithmic vertical scale to highlight the linear trend corresponding to exponential distribution. Non-normalized distributions for each VEI class, including error bars, are shown in Supplementary Fig. 1. The two panels show that the return times from the 2550 eruptions lying close to the approximately linear trends in Fig. 1 approximate exponential distributions (theoretical exponential distributions are reported as thick black lines). Normalization on the horizontal axis is done with respect to the mean return time for each VEI class, corresponding to the inverse of the rate parameter λ in Table 2. A value of 1 corresponds to the mean return time (or inverse of the rate parameter of the distribution) for each VEI class. Computed p-values (see Methods, and Supplementary Fig. 1) are >0.4 for all VEI classes, providing statistical robustness to the exponential distribution hypothesis. Colors of symbols: black = VEI 0; red = VEI 1; green = VEI 2; blue = VEI 3; turquoise = VEI 4; magenta = VEI 5; orange = VEI 6; indigo = VEI 7; maroon = VEI 8.

The dispersion of points around the linear trends in Fig. 1 is not a consequence of random, normally distributed errors or approximations. Rather, it reflects one fundamental character illustrated in Fig. 2 (and in Supplementary Fig. 1): within each VEI class, return times between subsequent eruptions are exponentially distributed. Maximum likelihood estimates (see Methods) of the rate parameters defining individual exponential distributions are reported in Table 2. For any given mean value in a distribution (in the present case, the mean return times, equal to the inverse of the rate parameters in Table 2), the exponential distribution is the one that maximizes entropy, meaning that it maximizes the dispersion of individual return times around the red dashed lines in Fig. 1. The richness of structures in each one of the cumulative plots of Fig. 1, with apparent clustering of eruptions followed by longer times of silence (equally present but not visible for small VEI classes at the scale of the plots), is fully reproduced by random exponential return times defined by the rate parameters in Table 2 (see Supplementary Fig. 2). Missing to account for the exponential distribution of return times can have relevant consequences, as one may misinterpret relatively long pauses as due to eruption under-recording, and/or focus the analysis on too short periods characterized by locally high/low eruption rates poorly representative of the long-term distribution.

**Table 2:** Maximum likelihood estimates (MLE) of rate parameters for the exponential distributions of return times.

VEI	MLE of rate parameters λ (day^−1^)	
	mean	variance
0	0.16401 × 10^−1^	0.67247 × 10^−6^
1	0.38609 × 10^−1^	0.25138 × 10^−5^
2	0.41752 × 10^−1^	0.17107 × 10^−5^
3	0.12473 × 10^−1^	0.51512 × 10^−6^
4	0.14904 × 10^−2^	0.24143 × 10^−7^
5	0.20026 × 10^−3^	0.97812 × 10^−9^
6	0.38165 × 10^−4^	0.42840 × 10^−10^
7	0.97473 × 10^−6^	0.22095 × 10^−13^
8	0.35126 × 10^−7^	0.45699 × 10^−16^

The rate parameters λ refer to the distributions in Fig. 2 (non-normalized distributions are shown in Supplementary Fig. 1). The exponential distributions have mean return times β equal to 1/λ, and variance β^2^.

As it is shown above, the quasi linear regions in Fig. 1 reflect a constant or stationary^23^ behavior, with the dispersion of data points revealing the exponential distribution of return times characterizing that behavior. The dispersion of data around the theoretical curves (in black) in Fig. 2 may reflect, instead, several elements. Statistical testing (see the Methods) suggests that most of the visible deviations are likely due to low number of data for large VEI classes; however, other factors may contribute, including i) uncertainties in eruption dating (see the Methods); ii) incompleteness in eruption records, although limited by the use of only data along the approximately linear regions in Fig. 1; and iii) uncertainties in estimated erupted volumes and unprecise assignment of VEI class. In order to avoid model-dependent artifacts, no attempts have been made to correct the rate parameters in Table 2. Those parameters reflect the data as they are, at the present level of knowledge of the eruption history of the Earth in the time windows identified by the approximately linear regions in Fig. 1. While it is expected that such a knowledge will evolve in the future, the quasi linear trends in Fig. 1, the close fit to exponential distributions in Fig. 2, and statistical tests reported in the Methods, concur to suggest that current knowledge limits are not such to destroy, mask, or substantially modify the effective distribution of eruption return times.

Exponential distributions naturally emerge when describing the length of inter-arrival times in homogeneous Poisson processes, suggesting that individual eruptions in the global database approximate completely random objects, in agreement with previous investigation^12,24–27^ on smaller datasets, or limited to large eruptions above a given size threshold. Conversely, other authors^28^ have claimed a log-normal distribution to describe a large subset of eruption repose times from the GVP database, when no distinction between eruption size classes is considered. Log-normal distributions require two fitting parameters instead of just one as for exponential distributions. They can approximate exponentially distributed quantities, except close to the vertical axis, where they bend down. As a consequence, their identification requires high confidence in the quality and representativeness of short to very short repose times in order to justify the introduction of one additional fitting parameter. The global database employed in this work does not show any apparent bending close to the vertical axis (Fig. 2, and Supplementary Fig. 1), not requiring anything more complex than a simple exponential distribution to represent the repose times of any VEI-scale eruptions at statistically significant levels of confidence (see the Methods).

Exponential distributions are memoryless, exactly meaning that the probability of observing a next event in a given time window is always the same, irrespective of the time passed from the last observed event. Therefore, the exponential distributions in Fig. 2 imply that when referring to the global scale, there is no “overdue” event: the probability of a next cataclysmic or colossal eruption somewhere on the Earth is always the same, no matter how long ago – one day or one million years – we observed the last one.

The exponential distributions in Fig. 2, with rate parameters from Table 2, constitute all of the information necessary to estimate the relative frequencies of the different VEI classes through Monte Carlo (MC) simulation (see Methods). Five million exponentially distributed random variates representing individual return times have been produced for each one of the nine VEI classes representing volcanic eruption magnitudes. Such a large number of MC runs ensure remarkable stability of the median and quantiles of the distributions pertaining to each VEI class; however, the mean frequencies of large VEI classes (≥6) do not converge to a stable quantity up to the largest number of MC runs performed (Supplementary Fig. 3). This observation is relevant, and it is further discussed below.

**Figure 3:**
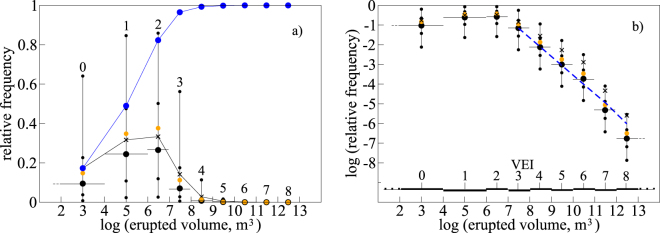
Relative frequencies of the different VEI class eruptions computed through MC simulation. For each VEI class (reported as a number above each distribution), the black circle represents the median of the MC distribution, and the smaller black circles represent the 50% and 90% confidence intervals; the x symbols represent the mean value after 5 million runs; the orange circles are frequencies directly obtained by the rate parameters λ in Table 2 (see Methods); and the blue circles are the cumulative of the mean values. The horizontal bars extend to cover the range of erupted volumes corresponding to each VEI class (Table 1). (**a**) Linear frequency axis. (**b**) Logarithmic frequency axis highlighting a power law (straight line) distribution of explosive eruption frequencies equal to or larger than VEI 3 (blue dashed line). The computed power law exponent is 1.95, with p-value = 0.65 providing statistical robustness to the power law hypothesis (see Methods).

Figure 3, and the Supplementary Table 1, show the results of the MC simulation. With reference to the mean values in panel a, about 50% of eruptions on Earth are of VEI 1 or lower, and more than 99% are of VEI 4 or lower. Panel b shows that in a log-log plot of frequency vs. erupted volume, explosive eruptions with VEI 3 or higher describe a power law behavior with exponent equal to 1.95. Extensive analysis, testing, sensitivity analysis and computational details related to the power law distribution of relative eruption frequencies is reported in the Methods.

### Implications of power law distributed explosive eruption frequencies

A power law distribution like the one in Fig. 3b, with exponent equal to 1.95, has substantial consequences for the overall eruptive dynamics at the Earth system scale. Power laws with exponent less than 2 have indefinite mean and variance (see Methods). In other words, with increasing number of observations neither the mean nor the variance reach a stable value: irrespective of how many samples we examine, they will continue to evolve with the sample population. One immediate consequence is that the question “What’s the mean explosive eruption on Earth” does not have any meaningful answer: simply, the mean continues to evolve as long as we add new information (or as long as the time goes by and new eruptions occur), due to the regular appearance of extremely large values as a consequence of indefinite variance. That situation (illustrated in Supplementary Fig. 4) provides an explanation to non-convergence of the mean values of frequencies for high VEI eruptions in the MC simulation (Supplementary Fig. 3), irrespective of how many MC runs (or how many observation) we add: extreme VEI eruptions continue to emerge, here and there, reflecting indefinite variance and continuously modifying the overall distribution. It is relevant to note that the current estimate of the power law distribution is close to 2. If a next improved estimate should bring that value above 2, then the mean would take a finite value, but the variance would still be indefinite (unless the power law exponent becomes larger than 3, which is by far a too large value compared to the available data). In such a case, volcanic eruptions would be capable of “black swan” behavior^29^: while the bulk of eruptions would define a stable mean, extreme eruptions would continue to emerge without any regularity, keeping the world under the unpredictable menace of a cataclysm with consequences comparable to those associated with the impact by a large celestial body.

Power law distributions emerge from many observations in fields as diverse as natural phenomena and man-made activities^29–32^. In geology, probably the most popular power law distribution is the Gutemberg-Richter law that describes the relative frequencies of earthquakes with different magnitude during a seismic crisis^33^. Power law distributions naturally originate in systems that are governed by highly non-linear dynamics with many degrees of freedom, when the system has evolved to a degree where complex behavior emerges in the form of scale-invariant processes ensuing from similar sets of conditions^31,34,35^. The paradigm is represented by the sand pile^34^: when a sand pile has evolved to a critical state such that no more sand can accumulate on top of it without rolling down, the size of avalanches resulting from further sand addition distributes as a power law, or as a linear trend in a log-frequency – log-size plot like the one in Fig. 3b. At such a critical state, each avalanche following a small disturbance, such as the addition of one single grain of sand, reflects exceedingly complex non-linear dynamics resulting from interactions between particles with many degrees of freedom, such as individual shape, position, angles, surface properties, etc. Scale invariance emerges as the macroscopic manifestation of similar fundamental processes and dynamics, a self-similar behavior that has its mathematical counterpart in power law distribution of the frequency of outcomes with orders of magnitude different size.

Non-linear processes with many degrees of freedom are known to govern volcano dynamics^36^. Critical conditions leading to scale-invariant behavior of volcanic processes have been described previously, involving duration of eruptions^37^, inter-eruption times^38–40^, magma fragmentation^41^, and microseismicity^42^. On a larger scale, explosive volcanic eruptions appear self-similar over several orders of magnitude: sub-Plinian VEI 3 eruptions, which occur repeatedly every year, are governed by the same fundamental processes, and show the same fundamental characteristics in terms of overall appearance, phenomenology, evolution, products, and geological records, of Plinian VEI 6 or VEI 7 eruptions, the latter having a mean return time of thousands of years (Table 2), and quite likely, of colossal VEI 8 eruptions that we have never observed in historical times and only know from their deposits. As a matter of fact, one could not distinguish small and large explosive eruptions by just looking at them, e.g., by comparing two pictures of volcanic plumes, in the absence of additional elements providing a scale to the observed phenomena. Together with power law distributed frequency of occurrence, such self-similarity is the fundamental feature of scale-invariant processes. The mechanisms and conditions governing from small to large explosive eruptions may therefore not differ in any of their fundamental characteristics; simply, the vast majority of situations end up with small eruptions, the same way as the vast majority of avalanches observed in a send pile are small.

## Conclusions

The return times of volcanic eruptions with different size, defined by the VEI scale, follow an exponential distribution, with rate parameters such that the relative frequencies of explosive eruptions with VEI ≥ 3 distribute as a power law with exponent close to 2. These findings support the conclusion that explosive eruptions from small to colossal, the former occurring repeatedly every year, and the latter never observed in historic times, are controlled by the same fundamental processes and conditions; and that highly impacting eruptions with VEI 7 or higher may occur any time, reflecting a distribution inherently characterized by the emergence of extreme, although rare, events. The above findings may have dramatic consequences for our forecasting capabilities: if the conditions leading to colossal eruptions with global, potentially disastrous impacts, are not fundamentally different from those associated to relatively small eruptions that we observe frequently, then discerning signs of their impending manifestation may be extremely difficult or impossible, at least at those many volcanoes where large eruptions occurred in the past, and in the absence of strong evidence of small amount of magma available for eruption. The science of volcanoes has enormously progressed during last decades, in terms of monitoring, modelling, measurements, experiments, and statistical analyses^43,44^. Notably, geophysical and geochemical records and sophisticated numerical modelling increasingly provide insights on deep conditions and processes below volcanoes^45,46^. It has been proposed recently that closed conduit volcanoes may show statistically significant increase in eruption size, at least up to VEI 5, with increasing length of repose time preceding the eruption^47,48^. It is however a matter of fact that volcanologists have not been able, to-date, to find any robust relationships between eruption size and multi-parametric signals collected during the pre-eruptive unrest. While that is perceived as one major limit of modern volcanology, the present results open the possibility that such a limit may represent a fundamental condition, rooted in exceedingly complex non-linear dynamics and exceedingly large degrees of freedom characterizing volcanic systems. Low-magnitude, relatively less impacting eruptions are by far more frequent; however, we may never be able to exclude the possibility that an impending eruption be of very high or extreme size, resulting in devastating effects, and impacting substantially at the global scale.

## Methods

### Eruption database and the use of the VEI scale as a measure of eruption size

The database in this work joins two open access databases constituted by the Global Volcanism Project (GVP) Holocene eruptions database of the Smithsonian Institution (http://volcano.si.edu), and the Large Magnitude Explosive Volcanic Eruptions (LaMEVE) database (http://www.bgs.ac.uk/vogripa). Both GVP and LaMEVE report the VEI scale when known. GVP refers to all eruptions in the Holocene (last ~12,000 years), while LaMEVE reports only eruptions with magnitude ≥ 4. In creating a unified database, I have summed up the eruptions in the two databases, and eliminated duplications. The resulting joined database contains 9517 individual eruptions with assigned VEI, spanning the entire VEI scale over a time extending back to more than 2 Ma; 2550 eruptions distribute along the approximately linear regions in Fig. 1, resulting in 2550 – 9 (VEI classes) = 2541 return times employed in the present analysis.

Several authors^11,16,17^ employ the Magnitude scale^15^, here indicated as M, as a measure of eruption size. M is determined by the logarithm of the erupted mass, espressed in kg, diminished by 7. The main advantage of M with respect to VEI is that it is a continuous scale, providing therefore a continuous representation of the size of volcanic eruptions. Furthermore, it refers to mass instead of volume (as for VEI), that being a more physically sound quantity, as the volume of the eruptive deposits depends on dynamic factors like efficiency of degassing and fragmentation, and on post-emplacement processes like welding and compaction. Thus, at least in principle, M should be preferred as a measure of eruption size. In practice, however, there are reasons that favor the use of VEI, that I briefly discuss in the following.

First of all, it must be noted that M is usually estimated starting from an estimate of the volume of the deposits. Therefore, both VEI and M are affected by the same errors and uncertainties at the basis of current-day methods to estimate eruption deposit volumes, and to estimate the proportion of the erupted magma that is dispersed over too wide regions to be retrieved by geological analysis. Estimating M also requires estimates of the distribution of density of the volcanic deposits, which adds additional uncertainty. Assigning a VEI class only requires an order of magnitude estimate of the erupted volume, while estimating M implies more refined knowledge of volumes and densities, which is hardly available. That results in crude estimates of M, often requiring grouping back into wide (up to one M unit, or one order of magnitude) classes in order to eliminate or smooth down spurious oscillations in the results^17^. It is shown^1,11^ that as a first approximation, an eruption with VEI x has a magnitude between x and x + 1. Grouping continuous M eruptions into discrete classes with width equal to one M unit means, *de facto*, referring to a scale analogous to the VEI scale (although selecting boundaries for M classes not coinciding with VEI boundaries^17^ blurs or destroys simple relationships between M and VEI).

The above discussion suggests that at the present level of knowledge, reference to M instead of VEI does not necessarily add to the accuracy of the analysis. Furthermore, out of the two employed GVP and LaMEVE databases, only the latter includes M estimates, and only for eruptions with M > 4 to which it exclusively refers; while VEI estimates are consistently^9^ included in both databases. Use of M instead of VEI implies therefore limiting the analysis to only eruptions with M > 4, which does not fit the objectives of this work.

Because of the many difficulties in quantifying eruption volumes, when estimating VEI volcanologists sometimes benefit from additional elements, such as plume height, or others that allow them to provide a VEI estimate based on their experience and expertise. That adds an element of uncertainty in VEI assignments. Especially for the very low 0, 1 VEI classes, the original relationships with volumes may be less traightforward, although that’s partly buffered by wider volume ranges associated with such classes (Table 1). In general, the need of just identifying an order of magnitude to define a corresponding VEI class reduces wrong assignments, although not eliminating them. Besides the above advantages and limitations, the use of VEI as a measure of eruption magnitude immediately communicates to volcanologists an order of magnitude accurate picture of eruption size and impacts, e.g., they immediately know what a VEI 6 (e.g., the Pinatubo eruption, Philippines, in 1991) means, and the differences with VEI 7 (e.g., the Tambora eruption, Indonesia, in 1815) or VEI 5 (e.g., the Puyehue eruption, Chile, in 2011).

### Eruption dating

Eruption dating comes from highly heterogeneous techniques encompassing stratigraphic, paleo-magnetic, and several radiometric methods^11^. Many data do not report any dating uncertainty. In such cases, I have adopted the following: for recent eruptions with historical accounts, the uncertainty has been set to zero if the eruption day is reported (the day constitutes in fact the time bit in this work), to 15 days when only the month and year are reported, and to 182 days when only the year is reported. Note that particularly for low to very low VEI eruptions, the database may report only the timing of eruption start, whereas the eruption peak may occur after significant time. Use of eruption start timing means referring to the start of the activity that leads to a peak activity corresponding to a certain VEI, rather than to the specific date of the peak activity. On the other hand, very low VEI eruptions are mostly associated with the formation of lava flows, with volumes that are commonly emplaced over long times corresponding to weeks, months, years, and in some cases (e.g., the current activity at Kilauea, Hawaii) tens of years. In all such cases, the date of the eruption in this work refers to the date of start of the eruption.

For ancient eruptions, and for eruptions with no historical accounts, I have first analyzed the 739 dating uncertainties reported in the LaMEVE database. The Supplementary Fig. 5 shows the distribution of dating uncertainties with time. On a careful inspection, the reported uncertainties are found to follow a log-normal distribution, shown as an S-shaped curve in the semi-logarithmic cumulative distribution in Supplementary Fig. 6. The Supplementary Fig. 7 shows the best-fit log-normal distribution of dating uncertainties (red curve), and the equivalent distribution reported on a linear horizontal axis (black line). The mean (log-normal) dating uncertainty is about 5%, with about 70% of the distribution between 2% and 16%. For the present aims, I have taken a 5% uncertainty as representative of all cases where the eruption date uncertainty is not reported, after having verified in preliminary tests that values in the 2–16% range did not produce appreciable differences; and have assumed that the uncertainty in the determination of each individual return time is given (as for normally distributed data) by the square root of the sum of the squares of the uncertainties in the corresponding eruption dates.

Return time uncertainties are reported in the panels of Supplementary Fig. 1. Large uncertainties in low VEI 0–2 classes reflect unspecified day or month of eruptions. Dating uncertainties are very low for VEI 3 eruptions, presumably because in the corresponding recent time range (back to AD 1950), significant impacts by VEI 3 eruptions catalyzed the attention of scientists and the civil society. For VEI 4 and 5 eruptions, their relatively short time windows (<2 and ~6 centuries, respectively), and return times measured in years, result in relatively small overall uncertainties. Uncertainties increase with VEI 6–8 eruptions, although they are overall smaller for larger VEI eruptions, presumably reflecting both more intense investigation on such extreme eruptions, and increased chances for more accurate dating as a consequence of more widespread dispersion of the volcanic products.

### Exponential distribution of eruption return times

A non-negative variable $x$ has an exponential distribution if it is distributed according to

1 $$p(x,\lambda )=\lambda {e}^{-\lambda x}$$

with complementary cumulative distribution function (CCDF)

2 $$F(x,\lambda )=1-{\int }_{0}^{x}p(x,\lambda )dx={e}^{-\lambda x}$$

where $p(x,\lambda )$ is the probability distribution function, and $\lambda$ is the rate parameter. The CCDF, employed in Fig. 2 and in Supplementary Fig. 1, describes the proportion of the distribution that lies above any specified value of $x$, and holds all of the properties of the original distribution without adding any bias from artificial grouping into classes. From equation (2) it follows that an exponentially distributed quantity plots as a straight line with slope $\lambda$ versus the logarithm of the CCDF (Fig. 2b, and right panels in Supplementary Fig. 1):

3 $$\mathrm{ln}\,[F(x,\lambda )]=\,\mathrm{ln}({e}^{-\lambda x})=-\,\lambda x$$

The maximum likelihood estimate (MLE) of the rate parameter $\lambda$ for a set of $n$ exponentially distributed data is given by:

4 $$\lambda =\frac{n}{{\sum }_{i=1}^{n}{x}_{i}}$$

Equation (4) has been employed to estimate the $\lambda$ parameters in Table 2, representing the mean eruption rate (number of eruptions per unit time). The inverse of $\lambda$ is therefore the mean return time ($\beta$ in equation (5) below).

The robustness of the exponential distribution hypothesis for eruption return times has been evaluated from Kolmogorov-Smirnov (KS) statistics, through the determination of the p-value. The p-value gives the probability that a random sample drawn from the hypothesized distribution, and having the same size of the observed sample, is at least as bad, or worse, than the observed sample in approximating the theoretical distribution. If the p-value tends to zero (in practice, if it is lower than a commonly accepted threshold of 5%), than it is unlikely that the observed data sample is truly drawn from the hypothesized distribution.

In order to estimate a p-value for each one of the nine VEI classes representing volcanic eruptions, I have drawn for each of them 1000 samples, each one with the same size of the observations (e.g., for VEI = 0, I have produced 1000 samples each one consisting of 399 return times, corresponding to 400 observed eruptions, see Fig. 1, and Supplementary Fig. 1). Random variates representing exponentially distributed return times $\beta$ have been generated from random homogeneous variates ${u}_{0;1}$ with uniform $(0;1)$ distribution:

5 $$\beta =\frac{-\mathrm{ln}({u}_{0;1})}{\lambda }$$

with $\lambda$ values from Table 2. Homogeneous variates ${u}_{0;1}$ have been generated with the ran2 package^49^ which implements L’Ecuyer random number generator with Bays-Durham shuffle, and which is thought to produce genuinely uncorrelated variates up to machine floating-points (a characteristic that is relevant to this work, where up to several million random numbers are generated).

The p-value for each VEI class is the proportion of the generated samples for which the maximum distance between their cumulative distribution and that of the theoretical exponential distribution is larger than the corresponding quantity computed from the observed sample:

6 $$p=\frac{1}{n}\sum _{i=1}^{n}i\,\ni \,\{{\rm{\max }}|{S}_{i}^{\text{'}}(x)-P(x)|\ge \,{\rm{\max }}|S(x)-P(x)|\}$$

where ${S}_{i}^{\text{'}}(x)$ and $S(x)$ are the distribution of the generated and observed samples, respectively, $P(x)$ is the theoretical exponential distribution with λ values from Table 2, and $n=1000.$ The computed p-values for each individual VEI class are reported in the corresponding panels in Supplementary Fig. 1; their high values confirm that exponential distribution of eruption return times is a robust hypothesis for the observed data.

### Monte Carlo simulation

Five million random exponentially distributed return times (equation (5)) for each one of the nine VEI classes have been produced. For each of them, the λ parameter has been randomly generated from a normal distribution with mean and variance from Table 2. Uniform $(0;1)$ variates have been generated as described above. For each of the 5 M sets of nine return times, the frequency of the i^th^ VEI class has been computed from each individual return time ${\beta }_{j}$ as:

7 $${f}_{i}=\frac{1}{{\beta }_{i}}{(\sum _{j=1}^{n}\frac{1}{{\beta }_{j}})}^{-1}{\rm{with}}\,{\rm{arithmetic}}\,{\rm{mean}}\,\widehat{{f}_{i}}=\frac{1}{m}\sum _{m}\,{f}_{i}$$

where $n=9$ (number of VEI classes) and $m=5M$ (number of MC runs). The quantiles of the distribution have been obtained, as usual, by the cumulative distributions of the ranked frequencies for each VEI class.

### Power law distribution of explosive eruption frequencies

A power law distribution is one of the general type

8 $$F(x)=\alpha {x}^{-k}$$

where $k$ is the scaling parameter (or power law exponent). Equation (8) diverges at increasingly lower values of $x$. That translates into the observation^31–34^ that practically no distributions in nature follow a power law to the smallest values of the variable $x$; at some value ${x}_{min}$ the power law breaks down, and equation (8) becomes:

9 $$F(x)=\frac{k-1}{{x}_{min}}{(\frac{x}{{x}_{min}})}^{-k}$$

Equations (8) and (9) result in a straight line with negative slope in a log-log plot like the one in Fig. 3b, where the logarithm of the frequency of each VEI class is plotted against the VEI number (or the logarithm of the erupted volume):

10 $$\mathrm{log}[F(x)]=\,\mathrm{log}(\frac{k-1}{{x}_{min}^{1-k}})-k\,\mathrm{log}\,x$$

Data placed along a straight line in a log-log plot, as in Fig. 3b, are a necessary signature of power law behavior, but they are, by far, not a sufficient condition^32,33^. Deciding whether an observed distribution is genuinely drawn from a power law is in fact not straightforward^32^. A general rule is that in the absence of additional evidence, the simplest functional form provided by the power law should be preferred^33^. Besides that simple argument, a test (described below) based on KS statistics confirms that power law distribution of eruption frequencies above VEI 3 is a robust hypothesis. That’s not a weak argument, as KS statistics have the tendency to over-reject even genuine (model-generated) power law distributions^33^. An additional argument (also developed below) supporting a power law distribution comes from the numerical experiment provided by the MC simulation: non-convergence of the arithmetic mean of high VEI classes is in fact consistent with a power law distribution with scaling parameter $\,k < 2$, as in the present case (see below).

In order to estimate the scaling parameter, the principles of linear regression applied to Eqs (8) or (9) are not adequate^32^, because power law distributed data do not hold any of the properties of normal distributions, characterized by a mean value and normally distributed errors (which is also the reason why visual alignment close to a straight line in a log-log plot does not demonstrate by itself a power law distribution). As a matter of fact, estimating the scaling exponent for power law distributed data is an open and active field of research^32^. Here I refer to the median of the frequency distribution for each individual VEI class, computed through MC simulation (Fig. 3). Being a measure of the central tendency of the distribution, the median is a stable quantity (Supplementary Fig. 3). The mean, instead, turns out to be largely unstable, especially for high VEI classes (Supplementary Fig. 3), as it strongly depends on extreme values in the distribution. For high VEI classes, and for exponentially distributed return times, such extreme values are orders of magnitude larger than the bulk of the distribution (Supplementary Fig. 1). A consequence of unstable mean is its progressive migration, with increasing VEI, towards the extremes of the distribution (see Fig. 3b). The Supplementary Fig. 4 provides a clear illustration of that.

In estimating $k$ it is relevant to consider that the frequencies in Fig. 3b are not directly observed quantities, being instead MC estimates; and that the VEI scale represents a discretization over a continuous distribution of erupted volumes (Table 1). The implemented procedure, based on KS statistics, is the following:

- Refer to the continuous distribution of erupted volumes, categorized into VEI classes in Table 1.
- Generate random, power law variates with exponent ${k}_{G}$, with the subscript $G$ indicating a guessed quantity. Power law variates above ${x}_{min}$ are produced according to

11 $$x={x}_{min}{u}_{0;1}^{\frac{1}{1-{k}_{G}}}$$

For each ${k}_{G}$, 10.8 million variates have been produced.

- For each distribution (each ${k}_{G}$), determine the frequency of VEI classes with $x\ge {x}_{min}$ by grouping the MC-produced erupted volume variates into their corresponding VEI classes. Then, evaluate the maximum distance $D$ between the cumulative distribution of the “data” (the MC-produced frequency distribution) and the model power law distribution with exponent ${k}_{G}$:
  12 $$D=\mathop{\max }\limits_{x\ge {x}_{\min }}|S(x)-P(x)|$$
  where $S(x)$ is the distribution of the data (MC frequencies), and $P(x)$ is the distribution for a power law with exponent ${k}_{G}$.
- The best estimate of *k* corresponds to the value of *k*_*G*_ that minimizes the distance $D$:

13 $$k={k}_{G}\,\ni \,D=\,{\rm{\min }}[\mathop{\max }\limits_{x\ge {x}_{\min }}|S(x)-P(x)|]$$

The above method results in a best choice of ${x}_{min}$ corresponding to VEI 3 (${x}_{min}={10}^{7}{m}^{3}$), for which $k=1.95$ is determined. That value has been used to draw the blue dashed line in Fig. 3b, which is therefore not – and it must not be – a simple best-fit of data points.

As for exponentially distributed return times, the robustness of the power law hypothesis for the distribution of VEI ≥ 3 eruption frequencies has been evaluated from KS statistics by determining a corresponding p-value. To this aim, a distance parameter $D$ (equation (12)) must be obtained for a large number of samples, each one representing randomly generated eruption frequencies with the same size of the observed sample.

In the present case, median frequencies in Fig. 3b (and Supplementary Table 1) are not observed quantities, rather, they result from MC simulation, with sample size (five million) which does not correspond to any observation. In order to estimate a p-value, relative frequencies $f$ of individual VEI classes have been estimated directly from the rate parameters $\lambda$ in Table 2:

14 $${f}_{i}=\frac{{\lambda }_{i}}{{\sum }^{}{\lambda }_{i}}$$

The orange symbols in Fig. 3a,b show relative frequencies calculated from equation (14). Frequencies calculated in this way do not describe the large variability embedded in exponential distributions of return times (Fig. 3), as they only relate to mean values. Figure 3 shows that such frequencies are close to median values; therefore, their distribution is not dissimilar from that of the median values, with the advantage that the frequencies from equation (14) are immediately drawn from the observed eruption data in Fig. 1. The procedure to estimate $k$ from the distribution of the median values of each VEI class distribution, described above, has been repeated to estimate an equivalent exponent for the distribution of the frequencies from equation (14). The size of the observed sample corresponds to the number of return times from which the $\lambda$ parameters in Table 2, for VEI ≥ 3 eruptions, were determined, and is equal to 533 (Fig. 1). With the estimated $k$ parameter, I have generated 1000 samples, each one constituted of 533 random power law variates, and for each sample, I have determined $D$ from equation (12). In 65% of cases (p-value = 0.65) that quantity was larger than the one from the distribution provided by equation (14). That result demonstrates that a power law hypothesis for the frequency distributions from equation (14) (orange symbols in Fig. 3b) cannot be rejected as a statistically robust hypothesis. Due to close similarity, I conclude that the same holds for the frequency distributions computed from MC simulation (black symbols in Fig. 3b).

### Sensitivity analysis

There is suggestion^16,17^ that VEI and M estimates in the database can be affected by systematic biases, e.g., systematic underestimation due to erosion and/or to difficulty in estimating the proportion of the erupted material in explosive eruptions which is taken by high atmosphere winds and dispersed over too wide regions to be confidently retrieved by geologic analysis or modelling. In order to evaluate the robustness of power law distributed relative eruption frequencies in Fig. 3b, I have conducted a sensitivity analysis. The analysis assumes that a given percentage, randomly sampled, of the data belonging to a specified VEI class, or specified group of VEI classes, is affected by systematic VEI underestimate, or systematic VEI overestimate, by one VEI unit. The results of the analysis are shown in Supplementary Fig. 8. Even with very high assumed percentage of mis-recording, in all cases the variability is contained within the range of variability of the distribution for each VEI class, when no biases are assumed. That result is likely a consequence of the order of magnitude differences in rate parameters associated with the different VEI classes (Table 2). Because the frequency of appearance of eruptions with different VEI is so large, systematic or selective biases involving even a relevant proportion of data do not largely affect the global distribution.

### Moments of a power law distribution

The ${n}^{th}$ moment of a power law distribution is given by

15 $$\begin{array}{rl}\langle {x}^{n}\rangle ={\int }_{{x}_{min}}^{\infty }{x}^{n}p(x)dx={x}_{min}^{n}(\frac{k-1}{k-1-n}) & {\rm{for}}\,k > n+1\end{array}$$

where $p(x)$ is the probability density function, the first moment ($n=1$) is the mean, and the second moment ($n=2$) is the variance of the distribution. From equation (15) it follows that when $k < 2$, as in the present case, the mean and the variance (and all higher order moments) of the distribution are not defined. In such a case, with increasing number of observations neither the mean nor the variance reach a stable value: irrespective of how many samples we are considering, they will continue to evolve with the sample population, due to the regular appearance of extremely large values as a consequence of infinite variance. That is consistent with the MC-produced mean values shown in the Supplementary Fig. 3: the mean never converges, irrespective of how many MC runs we add to the simulation (note that the median of the distribution is still defined when $k < 2$). Because this behavior is typical of power law distributions with exponent $k < 2$, the numerical results from the MC simulation provide one additional element supporting the conclusion that explosive eruption frequencies are distributed according to a power law.

## Electronic supplementary material

Supplementary Information
