# Supplementary material for: Global time-size distribution of volcanic eruptions on Earth
Source: Sci Rep. 2018 May 1;8:6838. doi: 10.1038/s41598-018-25286-y (PMC5931572; doi:10.1038/s41598-018-25286-y)
Supplement: Supplementary file 1 — Supplementary Information [file 41598_2018_25286_MOESM1_ESM.pdf]

Global time-size distribution of volcanic eruptions on Earth

Paolo Papale

Istituto Nazionale di Geofisica e Vulcanologia, Sezione di Pisa, via Della Faggiola 32, 56126 Pisa, Italy, paolo.papale@ingv.it

SUPPLEMENTARY INFORMATION

Supplementary Table 1. Monte-Carlo computed relative frequency distributions for the different VEI classes. The Monte Carlo simulation involves 5 million runs. The results are illustrated in terms of the mean and the quantiles of the corresponding distributions.

VEI	Mean	Percentiles				
		5th	25th	50th (median)	75th	95th
0	0.17233	0.76165×10^{-2}	0.37628×10^{-1}	0.94616×10^{-1}	0.22649	0.64044
1	0.31604	0.23187×10^{-1}	0.10779	0.24403	0.47456	0.84614
2	0.33353	0.26035×10^{-1}	0.11941	0.26567	0.50282	0.85995
3	0.14123	0.55611×10^{-2}	0.27508×10^{-1}	0.70169×10^{-1}	0.17472	0.56211
4	0.27756×10^{-1}	0.58547×10^{-3}	0.28723×10^{-2}	0.75291×10^{-2}	0.20644×10^{-1}	0.11359
5	0.53078×10^{-2}	0.77261×10^{-4}	0.37712×10^{-3}	0.98925×10^{-3}	0.27385×10^{-2}	0.16385×10^{-1}
6	0.12731×10^{-2}	0.14678×10^{-4}	0.71446×10^{-4}	0.18755×10^{-3}	0.52021×10^{-3}	0.31382×10^{-2}
7	0.47291×10^{-4}	0.37585×10^{-6}	0.18271×10^{-5}	0.47833×10^{-5}	0.13259×10^{-4}	0.80381×10^{-4}
8	0.23598×10^{-5}	0.13502×10^{-7}	0.65753×10^{-7}	0.17236×10^{-6}	0.47768×10^{-6}	0.28811×10^{-5}

Global time-size distribution of volcanic eruptions on Earth

Paolo Papale

Istituto Nazionale di Geofisica e Vulcanologia, Sezione di Pisa, via Della Faggiola 32, 56126 Pisa, Italy, paolo.papale@ingv.it

SUPPLEMENTARY INFORMATION

Supplementary Figure 1. Complementary cumulative frequency plots of eruption return times for each VEI class. Left panels: linear plots; right panels: logarithmic plots. Black open symbols: data; red lines: exponential curves with maximum likelihood rate parameters λ from Table 2. The exponential curve is a straight line with slope λ in the logarithmic plots at the right panels. Error bars are reported as gray lines (see Methods). For each VEI class, the corresponding p-value (see Methods) is reported.

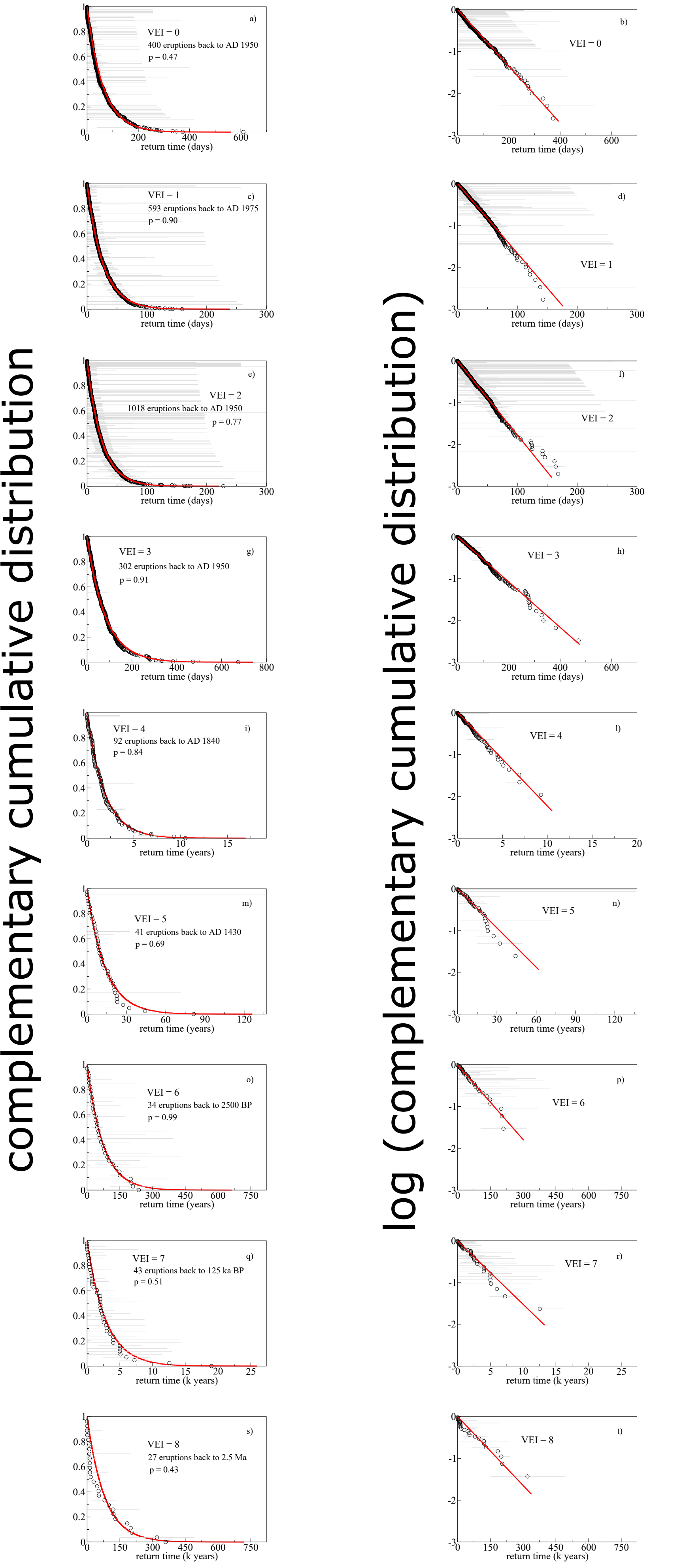
complementary cumulative distribution
log (complementary cumulative distribution)
a)
VEI = 0
400 eruptions back to AD 1950
p = 0.47
return time (days)
b)
VEI = 0
return time (days)
c)
VEI = 1
593 eruptions back to AD 1975
p = 0.90
return time (days)
d)
VEI = 1
return time (days)
e)
VEI = 2
1018 eruptions back to AD 1950
p = 0.77
return time (days)
f)
VEI = 2
return time (days)
g)
VEI = 3
302 eruptions back to AD 1950
p = 0.91
return time (days)
h)
VEI = 3
return time (days)
i)
VEI = 4
92 eruptions back to AD 1840
p = 0.84
return time (years)
l)
VEI = 4
return time (years)
m)
VEI = 5
41 eruptions back to AD 1430
p = 0.69
return time (years)
n)
VEI = 5
return time (years)
o)
VEI = 6
34 eruptions back to 2500 BP
p = 0.99
return time (years)
p)
VEI = 6
return time (years)
q)
VEI = 7
43 eruptions back to 125 ka BP
p = 0.51
return time (k years)
r)
VEI = 7
return time (k years)
s)
VEI = 8
27 eruptions back to 2.5 Ma
p = 0.43
return time (k years)
t)
VEI = 8
return time (k years)

Global time-size distribution of volcanic eruptions on Earth

Paolo Papale

Istituto Nazionale di Geofisica e Vulcanologia, Sezione di Pisa, via Della Faggiola 32, 56126 Pisa, Italy, paolo.papale@ingv.it

SUPPLEMENTARY INFORMATION

Supplementary Figure 2. Cumulative distribution of eruptions vs. time for various VEI classes. Black symbols: data; colored symbols: different sets of simulated distributions. The simulations assume exponential distribution of return times (Fig. 2 and Supplementary Fig. 1) with rate parameters λ from Table 2. For each simulated set, random normal variates for λ (Table 2), and random exponential variates for return times are produced, covering the time range of the data. The plots demonstrate that exponential distributions of return times fully explain the observed characteristics of the data, with apparent clustering of eruptions separated by longer silent times, and local variations of eruption rates. By contrast, panel b reports, as open black symbols, random normal variates of return times obtained by best-fitting the data for VEI 4 eruptions. Normal variates result in close-to-linear trends that do not reproduce the rich internal structures shown by the data.

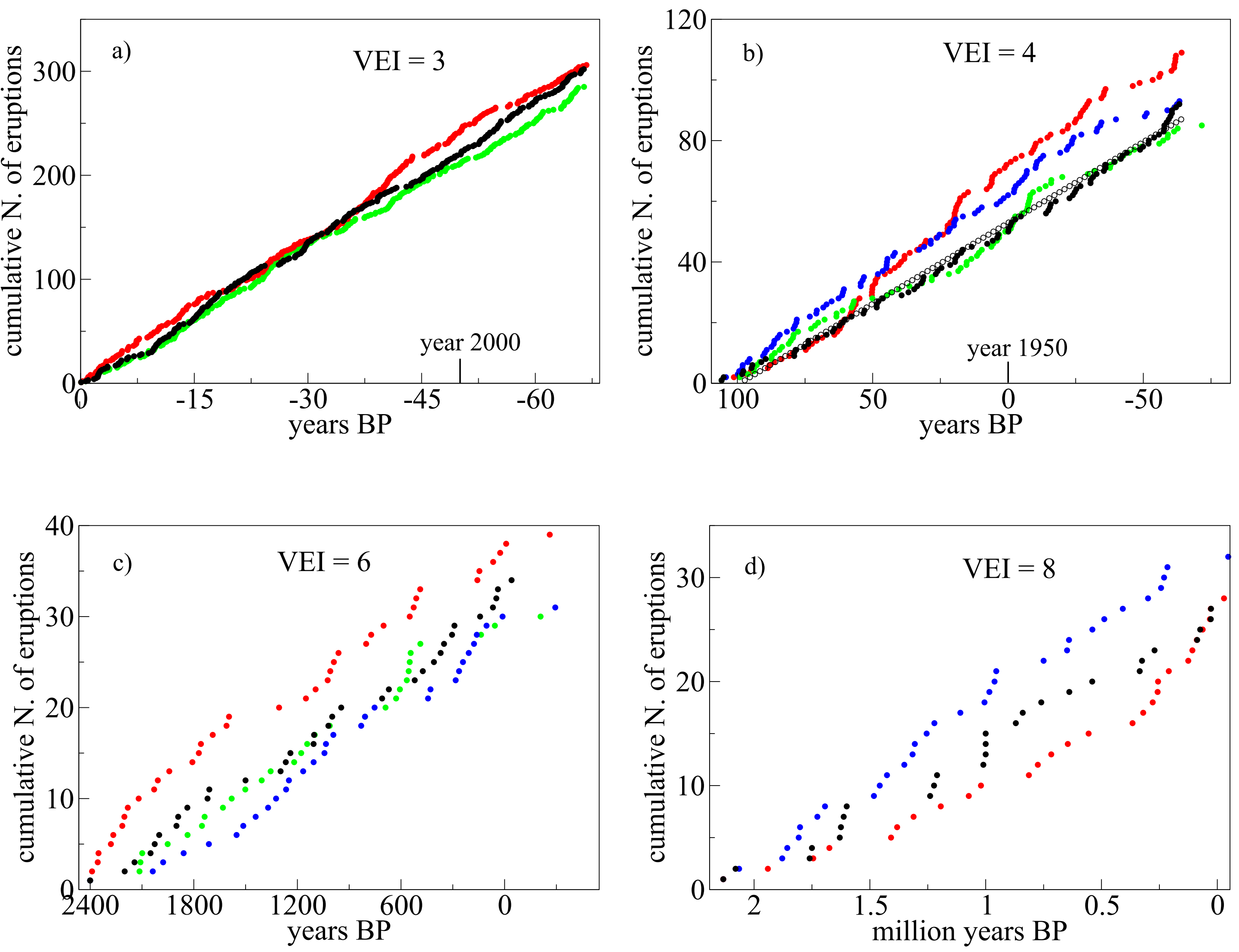

a)
VEI = 3
cumulative N. of eruptions
0
100
200
300
0
-15
-30
-45
-60
year 2000
years BP
b)
VEI = 4
cumulative N. of eruptions
0
40
80
120
100
50
0
-50
year 1950
years BP
c)
VEI = 6
cumulative N. of eruptions
0
10
20
30
40
2400
1800
1200
600
0
years BP
d)
VEI = 8
cumulative N. of eruptions
0
10
20
30
2
1.5
1
0.5
0
million years BP

Global time-size distribution of volcanic eruptions on Earth

Paolo Papale

Istituto Nazionale di Geofisica e Vulcanologia, Sezione di Pisa, via Della Faggiola 32, 56126 Pisa, Italy, paolo.papale@ingv.it

SUPPLEMENTARY INFORMATION

Supplementary Figure 3. MC simulations. The panels show, for each VEI class, the evolution of the arithmetic mean (a) and quantiles (b-d) of the distribution with increasing number of MC runs. The arithmetic mean (panel a) of high VEI classes show poor or no convergence until 5 million runs in the MC simulation. By contrast, the quantiles of the distribution in panels b-d, and more remarkably for the median values in panel b, converge well. Such trends reflect the exponential distribution of return times at the basis of the computations with the MC simulation, and the high sensitivity of the mean values to the extremes of the distribution (see also the Supplementary Fig. 4). Non-convergence of the mean for VEI ≥ 3 is also consistent with a power law distribution with scaling parameter < 2 (see the Methods). Colors as in Figure 2: black = VEI 0; red = VEI 1; green = VEI 2; blue = VEI 3; turquoise = VEI 4; magenta = VEI 5; orange = VEI 6; indigo = VEI 7; maroon = VEI 8.

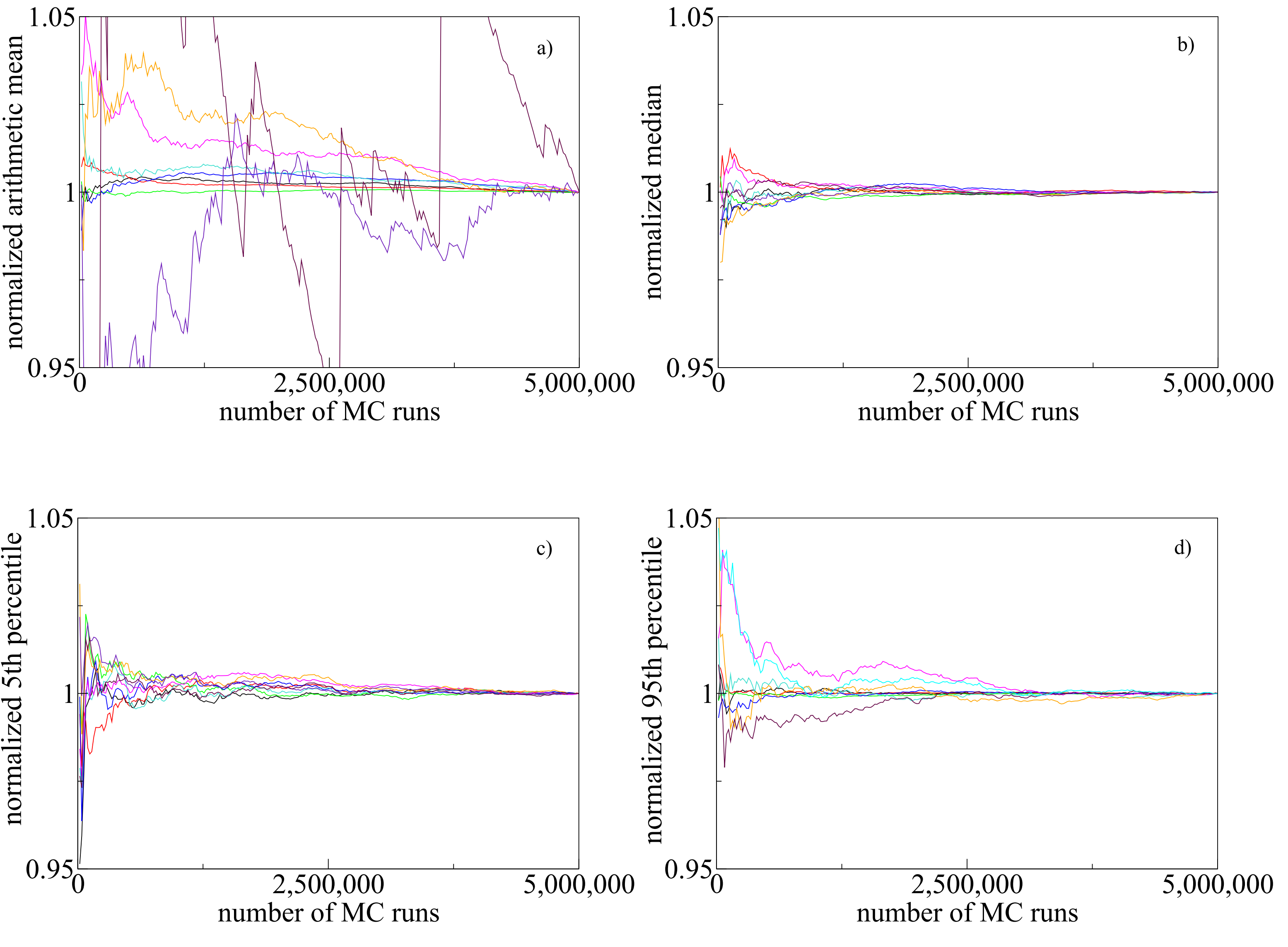
a)
b)
c)
d)
normalized arithmetic mean
normalized median
normalized 5th percentile
normalized 95th percentile
1.05
1
0.95
0
2,500,000
5,000,000
number of MC runs

Global time-size distribution of volcanic eruptions on Earth

Paolo Papale

Istituto Nazionale di Geofisica e Vulcanologia, Sezione di Pisa, via Della Faggiola 32, 56126 Pisa, Italy, paolo.papale@ingv.it

SUPPLEMENTARY INFORMATION

Supplementary Figure 4. Normalized frequency distributions from a MC simulation with 6000 runs. The figure illustrates the progressive migration, with increasing VEI, of the mean towards the upper extreme of the distribution. For each VEI class, normalization is done with respect to the mean return time, equal to the inverse of the corresponding λ parameter in Table 2. Mean value migration is a consequence of exponentially distributed return times, and of the orders of magnitude lower frequency of high VEI classes (Fig. 3 and Supplementary Table 1), whereby small oscillations in the computed frequency of low VEI classes cause orders of magnitude changes in high VEI frequencies (revealed in this figure by the right-twisted upper portion of the distributions for high VEI classes). The mean is largely affected by the extremes of the distribution, so that a large increase can be produced by a relatively small number of orders of magnitude higher frequencies. On the contrary, median values are largely insensitive to the extremes of the distribution. Colors as in Figure 2: black = VEI 0; red = VEI 1; green = VEI 2; blue = VEI 3; turquoise = VEI 4; magenta = VEI 5; orange = VEI 6; indigo = VEI 7; maroon = VEI 8.

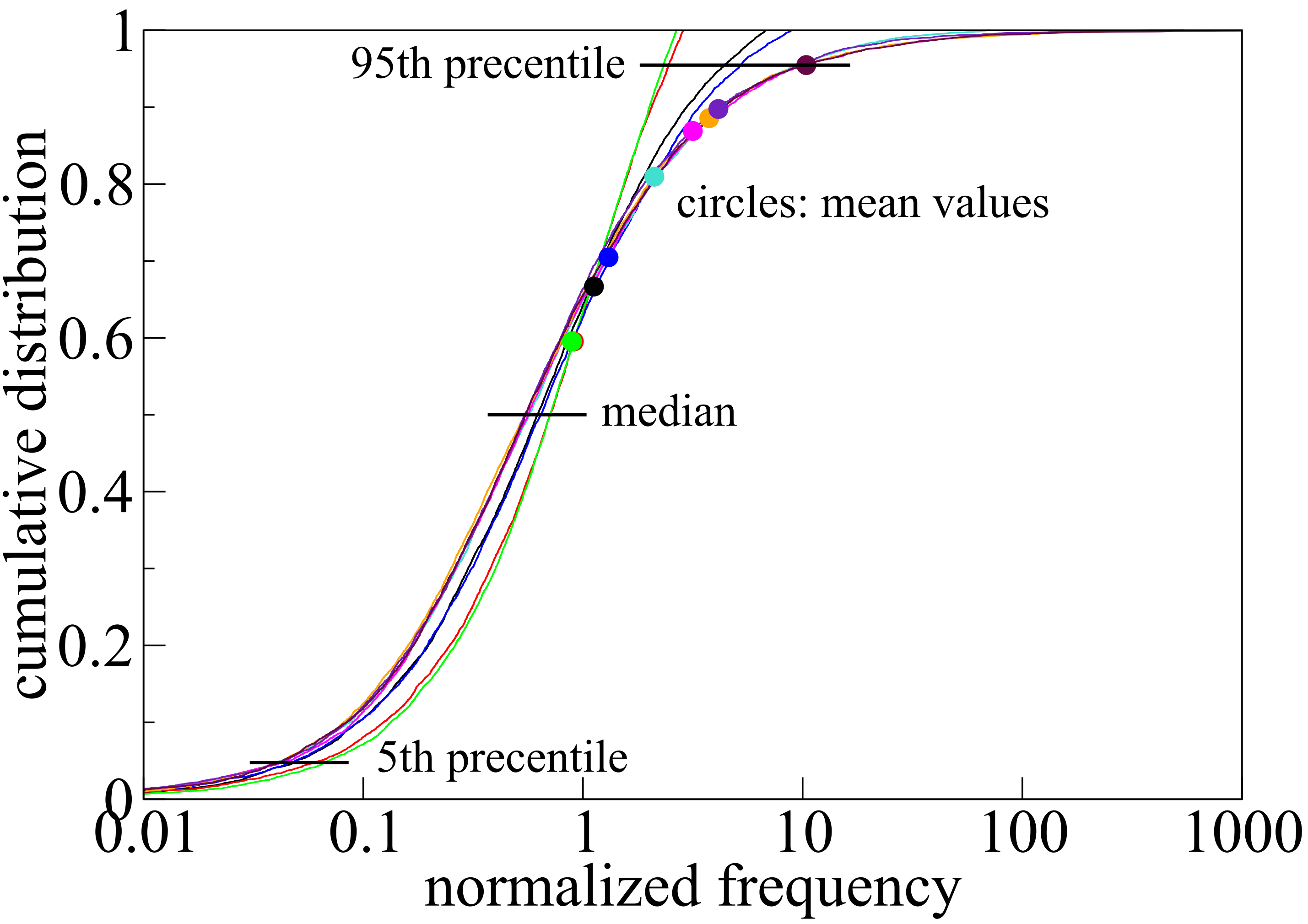

95th precentile
circles: mean values
median
5th precentile
cumulative distribution
normalized frequency
0
0.2
0.4
0.6
0.8
1
0.01
0.1
1
10
100
1000

Global time-size distribution of volcanic eruptions on Earth

Paolo Papale

Istituto Nazionale di Geofisica e Vulcanologia, Sezione di Pisa, via Della Faggiola 32, 56126 Pisa, Italy, paolo.papale@ingv.it

SUPPLEMENTARY INFORMATION

Supplementary Figure 5. Distribution of relative date uncertainty with time, for 739 eruptions from the LaMEVE database with reported date uncertainty.

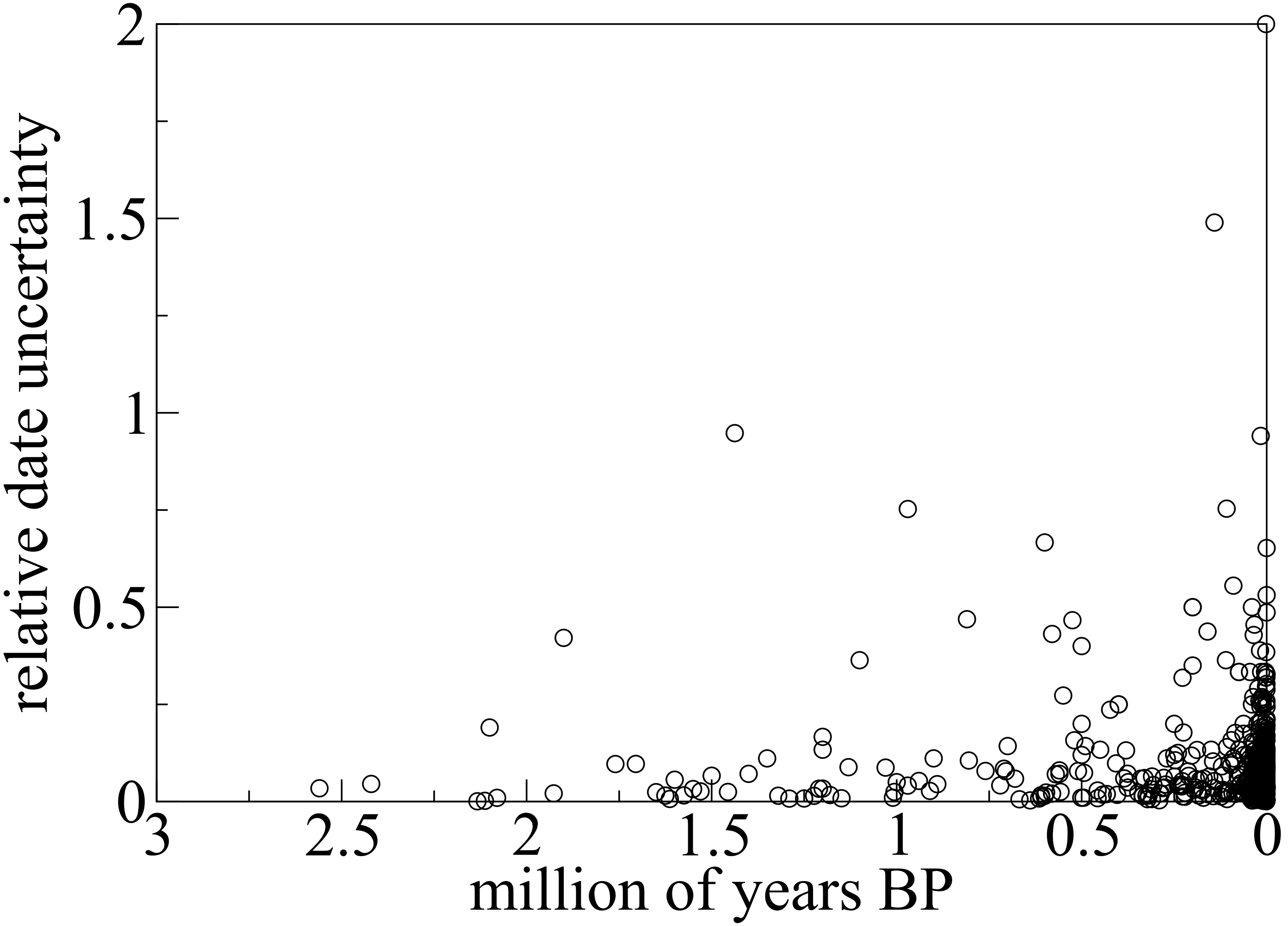

2
1.5
1
0.5
0
3
2.5
2
1.5
1
0.5
0
relative date uncertainty
million of years BP

Global time-size distribution of volcanic eruptions on Earth

Paolo Papale

Istituto Nazionale di Geofisica e Vulcanologia, Sezione di Pisa, via Della Faggiola 32, 56126 Pisa, Italy, paolo.papale@ingv.it

SUPPLEMENTARY INFORMATION

Supplementary Figure 6. Cumulative distribution of the frequency of the relative date uncertainty, for the data in Supplementary Fig. 5. Colors: black (thick line): all eruptions (739 data); blue (thick line): log-normal fit of all eruption data; turquoise: VEI 4 eruptions (292 data); magenta: VEI 5 eruptions (229 data); orange: VEI 6 eruptions (142 data); indigo: VEI 7 eruptions (62 data); maroon: VEI 8 eruptions (14 data). The left shift of the VEI 8 data points may reflect their low number and consequent poor representativeness, or in alternative, a true more accurate knowledge of eruption dates from more intensive investigation on colossal VEI 8 eruptions.

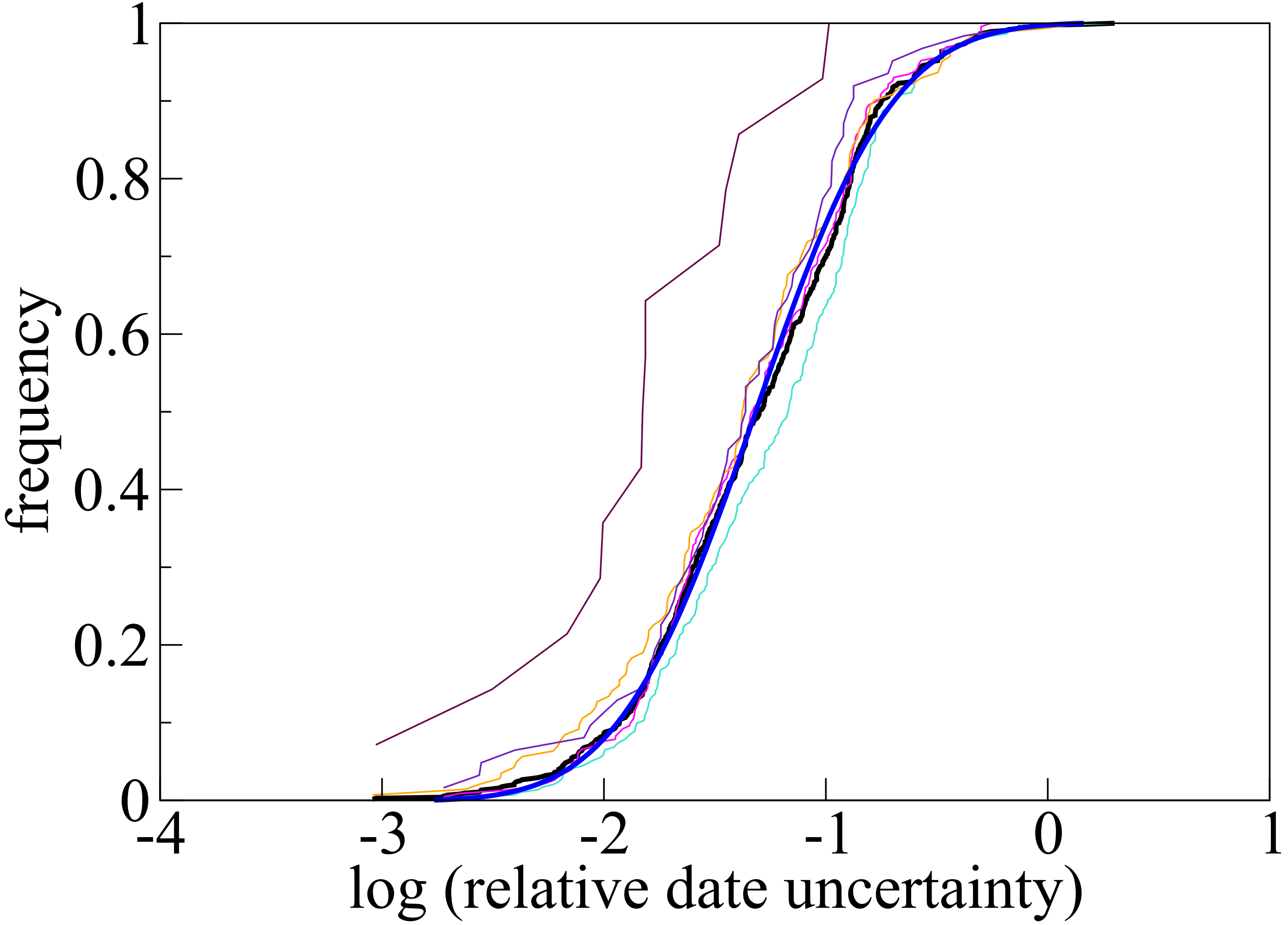

frequency
0
0.2
0.4
0.6
0.8
1
-4
-3
-2
-1
0
1
log (relative date uncertainty)

Global time-size distribution of volcanic eruptions on Earth

Paolo Papale

Istituto Nazionale di Geofisica e Vulcanologia, Sezione di Pisa, via Della Faggiola 32, 56126 Pisa, Italy,
paolo.papale@ingv.it

SUPPLEMENTARY INFORMATION

Supplementary Figure 7. Best-fit log-normal distribution of relative date uncertainties. The red and the black distributions are the same one, with the former plotted on a logarithmic horizontal scale, and the latter on a linear horizontal scale. The distribution of the data points is reported as open symbols along the horizontal axis (red: logarithmic scale; black: linear scale).

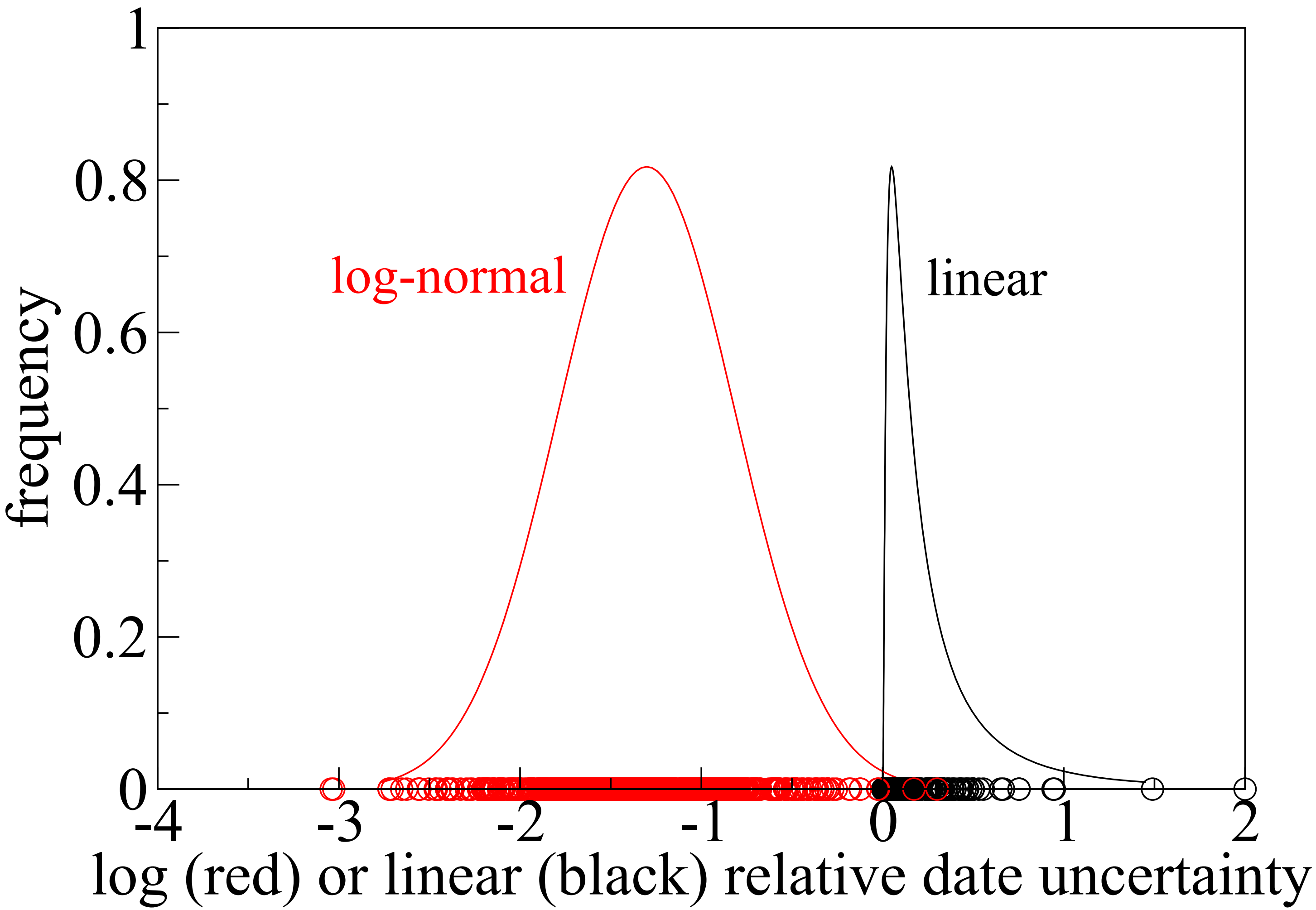

frequency
log-normal
linear
1
0.8
0.6
0.4
0.2
0
-4
-3
-2
-1
0
1
2
log (red) or linear (black) relative date uncertainty

Global time-size distribution of volcanic eruptions on Earth

Paolo Papale

Istituto Nazionale di Geofisica e Vulcanologia, Sezione di Pisa, via Della Faggiola 32, 56126 Pisa, Italy,
paolo.papale@ingv.it

SUPPLEMENTARY INFORMATION

Supplementary Figure 8. Sensitivity analysis on the occurrence in the database of systematic or selective biases in VEI assignment. Magenta symbols: systematic underestimation affecting all VEI classes (except VEI 8) by 10%, 20%, 30%, 40%, and 50% of the data in each VEI class. Green symbols: systematic overestimation affecting all VEI classes (except VEI 0) by 10%, 20%, 30%, 40%, and 50% of the data in each VEI class. Blue symbols: systematic underestimation selectively affecting the VEI classes from 2 to 4 by 10%, 20%, 30%, 40%, and 50% of the data in each VEI class. For each group of symbols above, progressive departure from the yellow symbol corresponds to progressively increasing percentage of mis-recording. All other symbols have the same meaning as in Fig. 3b.

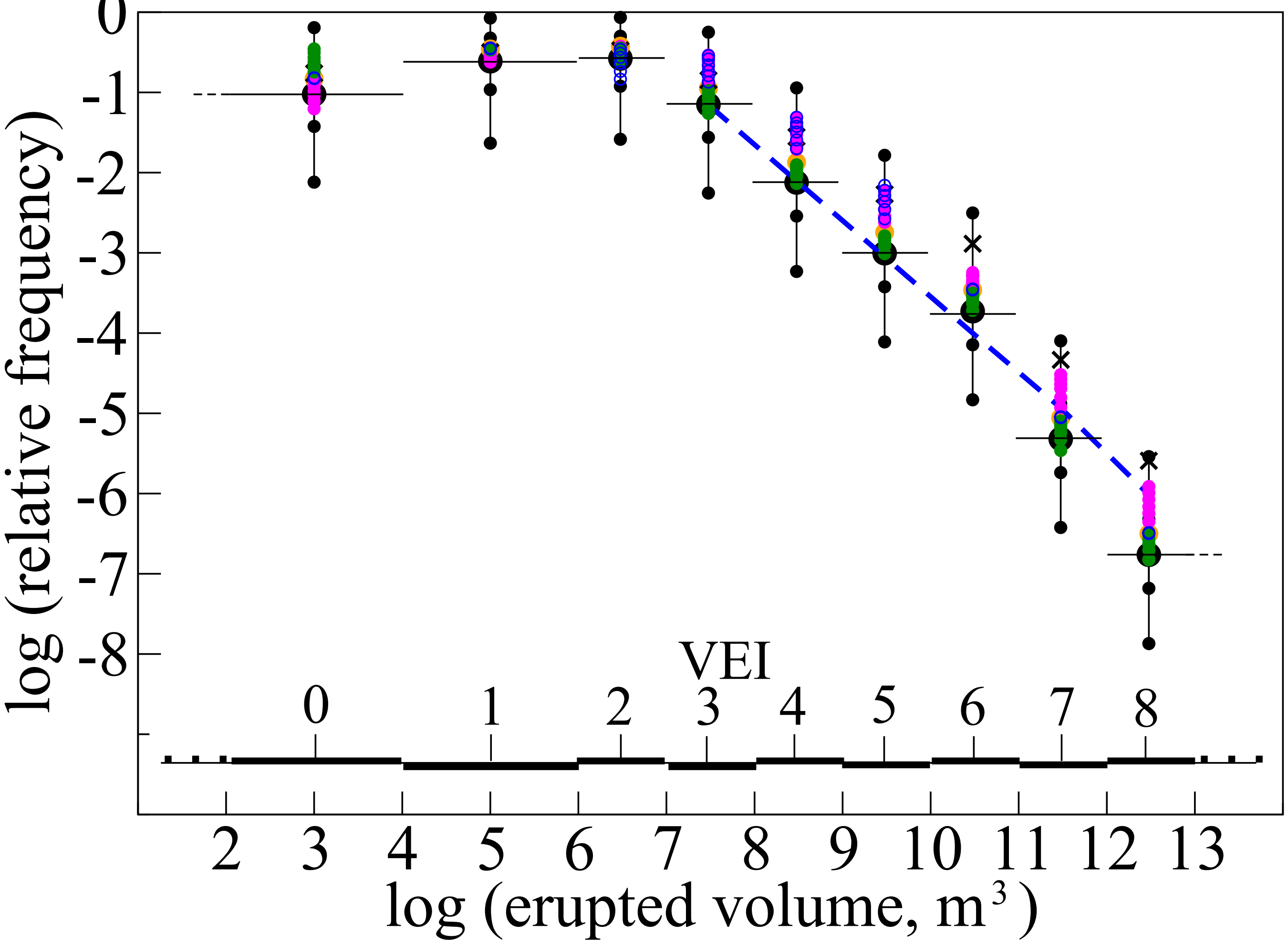

0
-1
-2
-3
-4
-5
-6
-7
-8
log (relative frequency)
VEI
0
1
2
3
4
5
6
7
8
2
3
4
5
6
7
8
9
10
11
12
13
log (erupted volume, m3)
